# Reliability of handheld autorefractometers in patients after cataract surgery

**DOI:** 10.55730/1300-0144.6078

**Published:** 2025-09-11

**Authors:** Ayça KÜPELİ ÇINAR, Abdulkadir Can ÇINAR, Hande GÜÇLÜ

**Affiliations:** Division of Anterior Segment, Cornea and Refraction, Department of Ophthalmology, Faculty of Medicine, Trakya University, Edirne, Turkiye

**Keywords:** Autorefractometer, cataract, measurements, refraction, reliability

## Abstract

**Background/aim:**

Accurate postoperative refraction assessment is essential for optimal visual rehabilitation after cataract surgery. Although handheld autorefractometers (HH-ARMs) are widely used in pediatric and uncooperative patients, their reliability in adults after cataract surgery remains uncertain. This study compared refractive outcomes obtained with a HH-ARM (Mediworks V100; Mediworks, Guangzhou, China) and a TB-ARM (Canon RK-F2; Canon Inc., Tokyo, Japan) to evaluate the clinical applicability of HH-ARM in this population.

**Materials and methods:**

This prospective study included 150 eyes of 150 patients (aged 55–85 years) who underwent uncomplicated phacoemulsification with intraocular lens (IOL) implantation. At 1 month postoperatively, spherical value (SV), cylindrical value (CV), cylindrical axis (CVA), and spherical equivalent (SE) were measured under cycloplegia with both devices. Agreement between devices was evaluated using nonparametric tests and Bland–Altman analysis.

**Results:**

SV and CV differed significantly between devices (p = 0.04 and p < 0.001, respectively), while no significant differences were observed in CVA (p = 0.44) or SE (p = 0.09). Bland–Altman analysis confirmed close agreement for SE (mean difference −0.08 D; 95% limits of agreement [LoA] −1.57 D to 1.42 D). Regression analysis indicated that SE values were statistically interchangeable, whereas SV and CV were not.

**Conclusion:**

HH-ARM demonstrated clinically acceptable agreement with TB-ARM for SE values, making SE the most reliable parameter for postoperative refraction in adults. These findings underscore the clinical relevance of HH-ARM as a practical alternative for patients with limited mobility and represent a novel contribution by validating its use in a population where evidence has been scarce. Further studies comparing HH-ARM with subjective refraction and across diverse surgical contexts are warranted.

## 1. Introduction

Automatic refractometers (ARMs), which play an important role in measuring refractive errors in contemporary eye examinations, have been used as an objective method since the early 1970s. These devices are easy to use and provide faster and more comfortable results for patients than manual retinoscopic refraction. Consequently, these devices have gained wide acceptance in clinical practice [1].

ARM measurements yield three primary parameters: spherical value (SV), cylindrical value (CV), and cylindrical axis (CVA). SV indicates whether the eye has clear vision at distance or near and quantifies the degree of refractive error in diopters (D) [2]. Positive (+) values indicate hyperopia, whereas negative (−) values indicate myopia [3]. CV determines the lens power required to correct astigmatism, a refractive error caused by irregular corneal or lenticular curvature, in diopters (D), and specifies its orientation through the CVA [4]. These parameters are fundamental for preparing spectacle and contact lens prescriptions. The spherical equivalent (SE) expresses refractive error in spherocylindrical prescriptions by incorporating astigmatism into the SV, yielding a single value. SE is calculated as SV + (CV / 2) [5]. This method is particularly useful when full astigmatism correction is unnecessary or when a simplified spectacle prescription is desired. This approach can improve patient comfort and simplify the prescription process. SE is also commonly used to assess refractive error when calculating intraocular lens (IOL) power after cataract surgery [6].

Accurate measurement of refractive error is essential for prescribing appropriate spectacles required for visual rehabilitation. Patients who receive accurately prescribed spectacles experience greater visual comfort in daily life and are better able to cope with the challenges associated with low vision. To prevent amblyopia in pediatric patients, it is necessary to identify and correct refractive errors [7]. Currently, various ARM devices are used for this purpose. In addition, handheld ARM devices have been developed that are noninvasive, easy to use, and provide rapid measurements, particularly for amblyopia screening in pediatric and disabled patients. Several clinical studies have compared these devices with retinoscopy and table-mounted ARMs (TB-ARMs). TB-ARMs are unsuitable for patients who are immobile, have limited mobility, poor cooperation, or spinal conditions such as ankylosing spondylitis. However, handheld ARMs (HH-ARMs), originally developed for pediatric populations, are also valuable for immobile or uncooperative adult patients [8–10].

Cataract is the leading treatable cause of blindness. Age-related cataracts account for approximately half of all visual impairments [11]. Modern phacoemulsification surgery is the standard treatment for cataract. Although refractive intraocular lenses are increasingly used, monofocal intraocular lenses remain the most common, and patients may still experience refractive errors postoperatively. Accurate postoperative refraction measurement and, when necessary, prescription of distance and/or near spectacles are essential for visual rehabilitation in these patients. TB-ARMs, which are commonly used today, cannot obtain measurements in patients with immobilization, poor cooperation, or restricted movement. Measurements obtained with pediatric HH-ARMs may be valuable in such patients. HH-ARMs allow measurement of both eyes simultaneously or separately from a distance of approximately 1 m [12].

Dik et al. [13] compared refraction measurements obtained with and without cycloplegia using a HH-ARM and a conventional ARM. They reported that the two devices showed consistency in cylindrical axis and SE measurements under cycloplegia, whereas SE measurements without cycloplegia were inconsistent between the devices.

TB-ARMs are commonly used in ophthalmology clinics. Obtaining measurements with a TB-ARM requires patients to sit and maintain head and neck stability, which is difficult for those with limited mobility, poor cooperation, or for children, making refraction unattainable in such cases. Retinoscopy is a time-consuming procedure, and its reliability is considered lower than that of ARMs [14]. HH-ARMs capable of remote measurement are gaining importance for these patients, and evaluating their measurement reliability is essential.

Although numerous studies have evaluated the accuracy of HH-ARMs in pediatric and uncooperative populations, evidence regarding their reliability in adults after cataract surgery remains limited. Since cataracts are generally age-related, many postoperative patients experience mobility or cooperation limitations that preclude the use of TB-ARM devices. However, little is known about whether HH-ARMs provide equally valid measurements in this population. To address this gap, this study compares residual refractive values obtained with the HH-ARM (Mediworks V100; Mediworks, Guangzhou, China) and the TB-ARM (Canon RK-F2; Canon Inc., Tokyo, Japan) in adults after cataract surgery, assessing the agreement between the two devices. This study aims to provide new evidence on the clinical applicability of HH-ARMs in adult populations and to support their potential role in visual rehabilitation strategies.

## 2. Materials and methods

Patients who underwent routine, uncomplicated cataract surgery for senile cataract in the Department of Ophthalmology, Faculty of Medicine, Trakya University and attended the 1-month postoperative visit were included. All patients underwent best corrected visual acuity (BCVA) testing with a Snellen chart, along with biomicroscopic and funduscopic examinations. Intraocular pressure (IOP) was measured in all patients.

After completing all examinations, pupils were dilated with a single installation of 1% cyclopentolate eye drops. After 30 min, residual postoperative refractive values were determined by two independent researchers using the TB-ARM (Canon RK-F2; Canon Inc., Tokyo, Japan) and the HH-ARM (Mediworks V100; Mediworks, Guangzhou, China), respectively. During HH-ARM measurements, the examiner stood at the patient’s eye level and at the distance indicated by the device. SV, CV, CVA, and SE values obtained from the two devices were compared.

Patient selection included individuals aged 55–85 years who underwent routine, uncomplicated phacoemulsification with intraocular lens (IOL) implantation for senile cataract. To standardize postoperative refractive stabilization, patients attending their 1-month follow-up visit were included. Exclusion criteria were applied to minimize confounding factors that might affect refractive measurements. These included previous ocular surgery or trauma, corneal surface abnormalities, media opacities affecting the optical axis, and refractive errors beyond the ±5 D measurement range of the HH-ARM. These exclusions ensured a more homogeneous study sample.

### 2.1. Statistical analysis

IBM SPSS Statistics version 26.0 (IBM Corp., Armonk, NY, USA) was used for statistical analysis. The Kolmogorov–Smirnov test was used to assess data normality. For continuous variables, numerical descriptive statistics included mean, standard deviation (SD), and minimum and maximum values. Frequencies and percentages were used for categorical variables. For comparing two dependent groups, data normality was first tested; as normality was not achieved, the Wilcoxon signed-rank test, a nonparametric method, was applied. Simple linear regression was performed to assess proportional bias between the two methods. A significance level of p < 0.05 was considered statistically significant.

## 3. Results

This study included 150 eyes of 150 patients who underwent uncomplicated cataract surgery. The mean age of the patients was 69.21 ± 6.7 years (range: 55–85). Of the patients, 79 (52.7%) were female and 71 (47.3%) were male. Among the eyes included in the study, 73 (48.7%) were right and 77 (51.3%) were left (Table 1).

Residual refractive values obtained with the TB-ARM and HH-ARM were compared. The suffix “T” was added to values obtained with the TB-ARM and “H” to those obtained with the HH-ARM. The mean SV-T was 0.05 ± 0.96 D (range: −2.50 to +4.75), the mean CV-T was −1.13 ± 0.88 D (range: −4.50 to +0.75), the mean CVA-T was 95.13° ± 37.74° (range: 0–180), and the mean SE-T was −0.50 ± 0.87 D (range: −3.25 to +3.00). The mean SV-H was −0.08 ± 0.86 D (range: −2.25 to +4.00), the mean CV-H was −0.70 ± 0.58 D (range: −2.75 to 0), the mean CVA-H was 93.18° ± 40.11° (range: 0–180), and the mean SE-H was −0.43 ± 0.85 D (range: −3.12 to +2.63). Comparison of TB-ARM and HH-ARM values showed statistically significant differences in SV (p = 0.04) and CV (p < 0.001), whereas no significant differences were observed in CVA (p = 0.44) or SE (p = 0.09) (Table 2).

**Figure 1:**
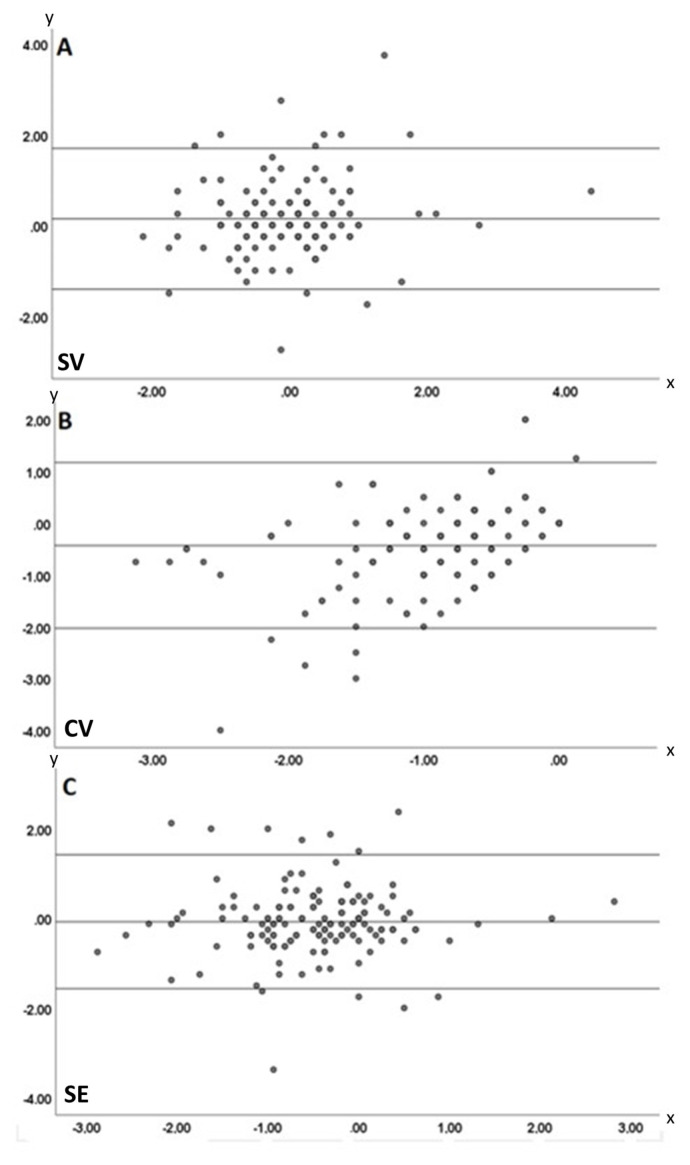
A) Bland–Altman plot for SV showing a mean (SD) difference of 0.14 D with 95% limits of agreement from −1.415 D to 1.695 D. B) Bland–Altman plot for CV showing a mean (SD) difference of −0.43 D with 95% limits of agreement from −2.033 D to 1.173 D. C) Bland–Altman plot for SE showing a mean (SD) difference of −0.0766 D (SD 0.30), with 95% limits of agreement from −1.566 D to 1.424 D.

Agreement between TB-ARM and HH-ARM measurements was further analyzed using Bland–Altman plots. For SV, the mean (SD) difference was 0.14 D, with 95% limits of agreement ranging from −1.415 D to 1.695 D (Figure 1A). For CV, the mean (SD) difference was −0.43 D, with 95% limits of agreement from −2.033 D to 1.173 D (Figure 1B). For SE, the mean (SD) difference was −0.077 D, with 95% limits of agreement from −1.566 D to 1.424 D (Figure 1C). Visual inspection of Bland–Altman plots indicated that HH-ARM produced values similar to TB-ARM on average across these three parameters. However, regression analysis demonstrated that only SE values were statistically comparable to TB-ARM, whereas SV and CV were not.

## 4. Discussion

This study aimed to compare refraction measurements obtained with TB-ARM and HH-ARM after cataract surgery. The findings indicated statistical differences between the two devices in SV and CV, whereas HH-ARM showed agreement with TB-ARM in SE and CVA. The difference in SV was modest (p = 0.0419), whereas the difference in CV was highly significant (p < 0.001). The observed difference in SV and CV, but agreement in SE, can be attributed to the fact that SE is a composite parameter calculated from SV and CV. Small but opposing shifts in SV and CV may offset each other, resulting in stable SE values. This suggests that SE is the most reliable parameter for clinical use when comparing HH-ARM and TB-ARM.

Uncorrected refractive errors are a major cause of visual impairment [15]. ARMs are easier to use, faster, and more reliable than other objective refraction techniques such as retinoscopy [16]. Previous studies have shown that refraction determined by ARM can be reliably compared with that determined by subjective refraction [17]. Although HH-ARMs were developed for pediatric use, they may also serve as an effective alternative to TB-ARMs for measuring refractive errors in adult patients who are unable to sit or cooperate [18]. These devices are available in various models and generally provide reliable results [15].

The findings of this study are consistent with previous reports. Liang et al. [19] reported that measurements obtained with the HH-ARM (Retinomax; Right Mfg. Co. Ltd., Tokyo, Japan) and the TB-ARM (Topcon KR-800; Topcon Corp., Tokyo, Japan) were consistent for CVA and SE. However, the Retinomax showed a tendency toward a myopic shift under noncycloplegic conditions. Castilla Martinez et al. [20] reported that HH-ARMs are consistent with TB-ARMs under cycloplegic conditions but show a hyperopic tendency under noncycloplegic conditions. Another study found strong correlations between the Nidek HH-ARM (Nidek AR K-30; Nidek Co. Ltd., Gamagori, Japan) and the TB-ARM (Huvitz HRK-7000A; Huvitz Co. Ltd., Anyang, South Korea) across all parameters of subjective refraction, suggesting that they can be used interchangeably [21]. In the present study, considering only the SV parameter, HH-ARM showed a myopic shift compared with TB-ARM, despite cycloplegia (HH-ARM mean: −0.08 D; TB-ARM mean: +0.05 D).

Prabakaran et al. [22] reported significant differences in SV and CV between HH-ARMs and TB-ARMs. In the present study, statistically significant differences were found in SV and CV, consistent with previous reports. Seymen et al. [23] compared refractive error measurements obtained with the Nidek HandyRef-K HH-ARM (Nidek Co. Ltd., Gamagori, Japan), Plusoptix A09 HH-ARM (Plusoptix GmbH, Nürnberg, Germany), Retinomax K-plus 3 HH-ARM (Right Mfg. Co. Ltd., Tokyo, Japan), and a TB-ARM, and found significant differences in SV, CV, and SE values. Handheld ARMs may yield different SE measurements compared with conventional desktop devices. For example, the Retinomax K-plus 5 (Right Mfg. Co. Ltd., Tokyo, Japan) has been shown to produce more hyperopic results in children [24]. Additionally, differences in SE measurements have been reported between the Nidek HH-ARM and desktop devices [21]. However, some studies have shown that SE measurements obtained with HH-ARMs are consistent with those from conventional desktop ARMs. For example, Retinomax K-plus 3 (Right Mfg. Co. Ltd., Tokyo, Japan) and Topcon KR-800 (Topcon Corp., Tokyo, Japan) have been reported to yield similar SE values under noncycloplegic conditions but different SE values under cycloplegia [20]. In the present study, all measurements were performed under cycloplegia to eliminate variability. No statistically significant difference was found between TB-ARM and HH-ARM for SE, but consistent with previous reports, HH-ARM yielded slightly more myopic results.

Sayed et al. [21] reported high similarity in SE and CV between sitting and supine positions with the Nidek HH-ARM, but noted differences in CVA. They suggested that this could be explained by significant cyclotorsion occurring in the supine position, particularly under monocular viewing conditions. Therefore, in this study, all HH-ARM measurements were performed in the sitting position at eye level, and CVA did not differ between TB-ARM and HH-ARM.

The results of this study indicate that HH-ARMs can serve as an alternative for adults with mobility limitations. Especially after cataract surgery, the use of HH-ARMs may be a reasonable option to improve patient comfort in those with mobility restrictions. However, while HH-ARMs provide acceptable accuracy for SV, CV measurements may differ substantially, making SE the most reliable parameter. Determining SE with HH-ARMs may be a suitable alternative for adult patients with limited mobility, patients with intellectual disabilities, and pediatric patients. SE is a practical and acceptable prescribing parameter for patients with low astigmatism [25]. This finding is clinically relevant for postoperative patients requiring spectacles. While discrepancies in SV and CV may lead to inaccurate prescriptions, the strong agreement in SE suggests that HH-ARMs can be safely used for functional visual rehabilitation, particularly in patients with limited mobility or cooperation.

Several recent studies support the findings of this study regarding the robustness of SE measurements. Karabulut et al. [26] reported excellent reliability for SE between TB-ARM and HH-ARM (mean difference: 0.11 D ± 0.47 D), despite modest changes in SV and CV, supporting the clinical stability of SE. Machine learning–enhanced prediction models developed by Turull-Mallofré et al. [27] achieved ±0.54 D agreement with subjective refraction for SE, reducing prediction error by 40% and offering promising avenues to improve the clinical applicability of objective measurements.

A major limitation of this study is the lack of a direct comparison with subjective refraction, which remains the gold standard for clinical decision-making. Without such comparison, the true interchangeability of HH-ARM and TB-ARM cannot be fully determined. Furthermore, this study included only postcataract patients aged 55–85 years, was conducted at a single center, and evaluated a single HH-ARM model. These factors may limit the generalizability of the findings.

Future studies should directly compare HH-ARM and TB-ARM with subjective refraction, include diverse patient groups such as those with premium or toric IOLs, and evaluate the long-term stability of measurements at multiple postoperative time points. Comparative studies among different HH-ARM models would also be valuable.

## Figures and Tables

**Table 1:** Demographic and clinical characteristics of the patients.

Parameter	Value
Mean Age (years)	69.21 ± 6.7(Min: 55, Max:85)
Sex	Female: 79 (52.7%)
	Male: 71 (47.3%)
Eye Laterality	Right: 73 (48.7%)
	Left: 77 (51.3%)

**Table 2:** Statistical analysis of measurements between TB-ARM and HH-ARM (median and range).

Parameter	TB-ARM(median, range)	HH-ARM(median, range)	Wilcoxon Test (p-value)
Spherical value (SV)	0.000 (−2.50 to (+4.75)	0.000 (−2.25 to +4.00)	p = 0.0419**
Cylindrical value (CV)	−1.00 (−4.50 to +0.75)	−0.50 (−2.75 to 0.000)	p < 0.001**
Cylindirical axis (CVA)	90.00 (0 to 180)	90 (0 to 180)	p = 0.4480
Spherical equivalent (SE)	−0.50 (−3.25 to +3.00)	−0.375 (−3.12 to +2.63)	p = 0.0946

* TB-ARM; table-mounted-autorefractometer, HH-ARM; handheld autorefractometer.

** p < 0.05.
